# Supplementary material for: Splice-Junction-Based Mapping of Alternative Isoforms in the Human Proteome
Source: Cell Rep. Author manuscript; Available in PMC 2020 Jan 15. (PMC6961840; doi:10.1016/j.celrep.2019.11.026)
Supplement: 2 [file NIHMS1546469-supplement-2.zip › DF1/PXD010154/Heart/Heart_19_TTN_SNCTVSVHVSEHEVPPFFDLKPVSVDLALGESGTFK.pdf]

A

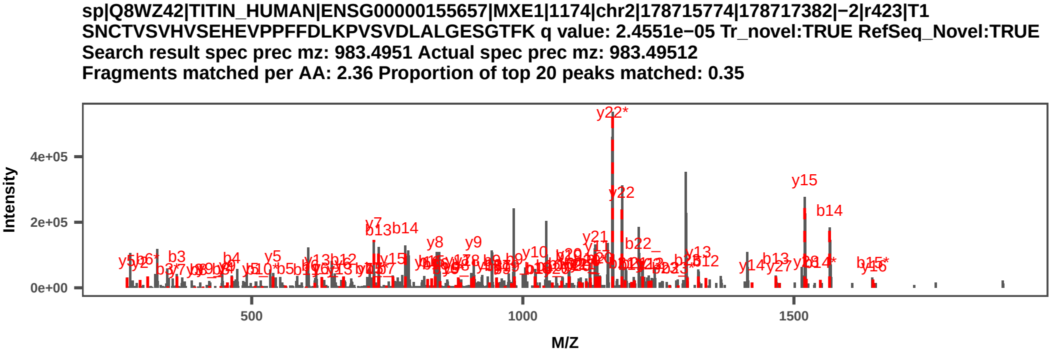
sp|Q8WZ42|TITIN_HUMAN|ENSG00000155657|MXE1|1174|chr2|178715774|178717382|−2|r423|T1
SNCTVSVHVSEHEVPPFFDLKPVSVDLALGESGTFK q value: 2.4551e−05 Tr_novel:TRUE RefSeq_Novel:TRUE
Search result spec prec mz: 983.4951 Actual spec prec mz: 983.49512
Fragments matched per AA: 2.36 Proportion of top 20 peaks matched: 0.35
Intensity
4e+05
2e+05
0e+00
M/Z
500
1000
1500
y22*
y22
y15
b14
y8
y9
y21
y13
b3
b4
y5

B

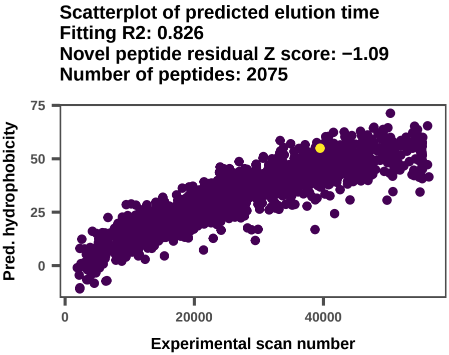
Scatterplot of predicted elution time
Fitting R2: 0.826
Novel peptide residual Z score: −1.09
Number of peptides: 2075
Pred. hydrophobicity
75
50
25
0
0
20000
40000
Experimental scan number

C

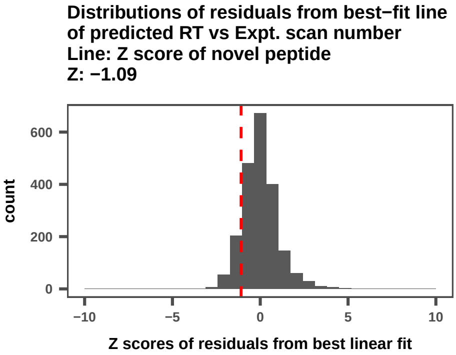
Distributions of residuals from best−fit line
of predicted RT vs Expt. scan number
Line: Z score of novel peptide
Z: −1.09
count
600
400
200
0
−10
−5
0
5
10
Z scores of residuals from best linear fit
